# Effect of feeding varying levels of groundnut haulms on feed intake and growth performance in broiler chickens

**DOI:** 10.14202/vetworld.2015.139-142

**Published:** 2015-02-09

**Authors:** N. K. Ribadiya, H. H. Savsani, S. S. Patil, D. D. Garg, M. R. Gadariya, V. K. Karangiya, A. P. Gajera

**Affiliations:** 1 Department of Animal Nutrition, College of Veterinary Science and A. H., Junagadh Agricultural University, Junagadh, India; 2 Department of Animal Nutrition, Intensive Livestock Farm Complex, College of Veterinary Science and A. H., Junagadh Agricultural University, Junagadh, India; 3 Department of Animal Nutrition, Cattle Breeding Farm, Junagadh Agricultural University, Junagadh, India

**Keywords:** body weight, broiler chickens, feed intake, groundnut haulmsIntroduction

## Abstract

**Aim:**

This study was carried out to evaluate groundnut haulms (GNH) as alternate feed source by its incorporation and assessment in terms of feed intake and growth performance in the diets of broilers.

**Materials and Methods:**

A total of 240 1-day-old Cobb-400 broiler chicks were randomly assigned to four dietary treatments each with three replicates (n=60). Experimental Birds in group T1 were fed with conventional feed while birds in T2, T3, T4 were fed containing 2%, 4%, and 6% of GNH replacing maize and soyabean on iso-nitrogenous basis.

**Results:**

Feed intake increases significantly (p>0.05) with increasing level of GNH in the diets of experimental birds. Highest feed intake was recorded in T4 (6% GNH), followed by T3 (4% GNH) than T2 (2% GNH) and T1 (control). Birds fed GNH gained significantly (p<0.05) higher body weight than birds fed the control diet. Birds in T4 [6% GNH] gained highest body weight, followed by T3 (4% GNH) than T2 (2% GNH) and T1 (control). However, feed conversion ratio (FCR) remained non-significant for all treatment groups.

**Conclusion:**

On the basis of the results of this study, it is concluded that supplementation of GNH can successfully replace costly ingredients like maize and soybean meal in the diets of broiler birds up to the level of 6 percent of concentrate mixture without any harmful effects on feed intake, growth and FCR.

## Introduction

Over a period due to the combined efforts of government, private players, and farmers, the poultry industry has become a full-fledged organized sector and now stands as one of the fastest growing industries in India. Poultry occupies an important place in Indian economy by contributing more than Rs. 11,000 crores to the national Gross Domestic Product. It also ranks 3^rd^ and 5^th^ with respect to production of egg and meat in the world [1]. However, a major constraint to poultry production in India is the very high cost of conventional feeding stuff and therefore cost of poultry production has gone up substantially in recent years [2]. Since feed cost is a major expense in poultry production, accounting for 60-70% of total production cost [3], search for cheap, locally available and equally nutritive feed source to partially substitute commercial poultry diet is ever increasing [4,5].

Leaf meal is one of the alternative feed, which has been incorporated in the diets of poultry as a means of reducing the high cost of conventional protein sources [6]. There is evidence in the literature of the beneficial effects of using leaf meal from different sources in poultry production [7-9]. Ground nut (*Arachis hypogaea*) haulms (GNH) are the residue left after harvesting groundnut and are produced at very high quantity in Saurashtra, which can be used in a similar way to different leaf meals in the diet of poultry. It is a good source of protein and calcium [10] and is referred in vernaculars language as guitar. GNH have good nutritive value and contains about contain 12.4% crude protein (CP) on dry matter (DM) basis [11]. Although abundant research has been carried out on feeding GNH in ruminants very few information is available on feeding GNH in poultry.

Hence, the present study has been carried out to assess the effect of feeding varying levels of GNH on feed intake and growth performance in broiler birds.

## Materials and Methods

The experiment was carried out at the Shambhu poultry farm at Dhoraji, Dist. Rajkot, Gujarat, India.

### Ethical approval

This research was carried out after approval of Institutional Animal Ethics Committee.

### Source and processing of GNH

GNH used in this study was bought from Cattle Breeding Farm, Junagadh Agricultural University, Junagadh.

### Experimental birds and diets

Day old 240 broiler chicks of cobb-400 strain with average body weight 45.17-46.47 g were wing banded and distributed randomly into four groups having three replicates of 20 birds each by randomized block design and allocated to four dietary treatments as T1, T2, T3 and T4 using to the four experimental diets. Experimental Birds in group T1 were fed with conventional concentrate mixture while birds in T2, T3, T4 groups were fed with concentrate mixture containing 2%, 4% and 6% of GNH replacing maize soyabean on iso-nitrogenous basis. Ingredient and chemical compositions of these starter and finisher rations are presented in Table-1 and -2 respectively. Chemical composition of GNH is given in Table-3.

**Table-1:** Ingredient and chemical composition of starter feeds.

	T1	T2	T3	T4
Feed ingredient (kg)				
Maize	59	58	56	54
Soyabean	36	35	35	35
GNH	0	2	4	6
Fat/oil	2	2	2.3	2.7
Lime stone	0.8	0.8	0.8	0.8
Di calcium phosphate	1.7	1.7	1.7	1.7
Salt	0.35	0.35	0.35	0.35
Methionine	0.11	0.11	0.11	0.11
Lysine	0.15	0.15	0.15	0.15
Trace minerals	0.0625	0.0625	0.062.5	0.0625
Vitamin premix	0.050	0.050	0.050	0.050
Toxin binder	0.1	0.1	0.1	0.1
Total	100.00	100.00	100.00	100.00
Chemical composition (% DM basis)				
ME (Kcal)	3020.5	2999.0	2995.0	2966.5
DM	95.52	95.99	95.99	95.99
OM	90.4	90.32	89.98	89.58
CP	21.96	21.90	21.83	21.77
CF	3.65	4.10	4.45	5.16
EE	3.33	3.80	3.92	4.00
NFE	61.46	60.52	59.78	58.65
Total ash	9.60	9.68	10.02	10.42
Silica	0.85	1.52	1.64	1.76
Calcium	1.02	1.01	1.00	0.99
Phosphorus	0.45	0.45	0.46	0.47

ME=Metabolic energy, DM=Dry matter, OM=Organic matter, CP=Crude protein, CF=Crude fiber, NFE=Nitrogen free extract, GNH=Groundnut haulms, EE=Ether extract

**Table-2:** Ingredient and chemical composition of finisher feeds.

	T1	T2	T3	T4
Feed ingredient (kg)				
Maize	65	65	65	65
Soyabean	30	28	26	24
GNH	0	2	4	6
Fat/oil	2	2	2	2
Lime stone	0.8	0.8	0.8	0.8
Di calcium phosphate	1.7	1.7	1.7	1.7
Salt	0.35	0.35	0.35	0.35
Methionine	0.11	0.11	0.11	0.11
Lysine	0.18	0.18	0.18	0.18
Trace minerals	0.0625	0.0625	0.062.5	0.0625
Vitamin premix	0.050	0.050	0.050	0.050
Toxin binder	0.1	0.1	0.1	0.1
Total	100.00	100.00	100.00	100.00
Chemical composition (% DM basis)				
ME (Kcal)	3077.5	3069.5	3061.5	3053.5
DM	95.54	95.98	95.99	95.99
OM	90.38	90.32	89.90	88.76
CP	19.89	19.35	18.92	18.56
CF	3.44	3.87	4.43	5.01
EE	3.35	3.77	3.82	4.02
NFE	63.70	63.33	62.73	61.17
Total ash	9.62	9.68	10.10	11.24
Silica	0.84	1.45	1.58	1.70
Calcium	0.96	0.97	0.99	1.00
Phosphorus	0.43	0.45	0.45	0.44

ME=Metabolic energy, DM=Dry matter, OM=Organic matter, CP=Crude protein, CF=Crude fiber, NFE=Nitrogen free extract, GNH=Groundnut haulms, EE=Ether extract

**Table-3:** Chemical composition of GNH (In % DM basis).

DM	97.83
OM	74.64
CP	9.82
CF	27.80
EE	2.97
NFE	34.05
Total ash	25.36
Calcium	2.94
Phosphorus	0.31
Silica	13.14

DM=Dry matter, OM=Organic matter, CP=Crude protein, CF=Crude fiber, NFE=Nitrogen free extract, GNH=Groundnut haulms, EE=Ether extract

### Feeding and management procedures

All the experimental birds were housed in a well-ventilated shed in deep litter pens under uniform managerial conditions. The reference and test concentrate mixtures and clean drinking water was supplied to the birds *ad libitum* throughout the study period to meet the nutrient as per [12]. The body weights of all the birds were recorded weekly in the morning, before feeding and watering. The amount of feed offered and residue left after 24 h was measured to find out feed intake of feed. Feed conversion ratio (FCR) was calculated by dividing the feed intake by weight gain. Vaccination and other routine poultry management practices were carried out neatly.

### Chemical and statistical analysis

Samples of feeds were milled to pass through a 1mm sieve and then analyzed following the methods of AOAC (1995) to determine DM by the oven drying method (934.01), organic matter by muffle furnace incineration (967.05), CP by kjeldahl method (984.13) (n×6.25), ether extract (EE; 920.39), ash (942.05). The data collected on various parameters were analyzed using the method [13].

## Results and Discussion

### Nutrient composition of GNH and experimental rations

Nutrient composition of GNH and different concentrate mixtures are given in Table-1. The GNH contains about 9.82% CP and 34.05% nitrogen free extract, and its nutrient composition was comparable to values reported by earlier workers [6]. All the feeds were iso-nitrogenous and comparable with respect to their proximate composition.

### Feed intake (g/bird)

The average total feed intake showed a significant difference (p>0.05) among different dietary treatments (Table-4). Feed consumption of birds in different groups was increased significantly (p>0.05) with increased inclusion level of GNH. The highest feed consumption was recorded in birds fed T4 diet, followed by T3, T2 and T1 group. Inclusion of higher levels GNH from 0 to 6 percent (from T1 to T4 groups) has also leads to linear increase in crude fiber content of different ration which might be along with lower dry matter digestibility are responsible for increase feed intake on higher level of supplementation of GNH in broilers [14]. Reported similar findings on feeding *Moringa oleifera* leaf meal (MOLF), *Acacia angustissima* and *Moringa stenopetala* leaf meal respectively in the diets of broilers [6,15,16].

**Table-4:** Performance of experimental birds.

Attributes	Treatments				SEM

	T1	T2	T3	T4	
Body weight (kg)					
Initial	45.80	45.17	46.47	45.58	0.30
Final	1376.88^d^	1422.00^c^	1526.78^b^	1596.82^a^	13.63
Average final body weight gain (g)	1331.08^d^	1376.84^c^	1480.31^b^	1551.23^a^	25.38
Average weekly weight gain (g)	210.35^b^	213.55^b^	234.00^a^	245.23^a^	3.69
Average total feed intake	2684.22^d^	2808.99^c^	3026.65^b^	3268.39^a^	40.37
Feed conversion ratio	1.96	1.95	1.98	2.00	0.01

Means with different superscript differ significantly (p<0.05), SEM=Standard error of mean

### Body weight gain (g) and FCR

Total body weight gain (g) and weekly body weight gain (g) for the period of 42 days were significantly (p<0.001) higher in GNH supplemented groups (T2, T3 and T4) than control (T1) group with increasing trend from T1-T4 groups (Table-4). While, FCR (kg DMI/kg gain) of the experimented birds showed non-significant difference among different groups. Improvement in body weight in treatment groups T4, T3 and T2 might be due to the better feed intake and nutrient availability as GNH is a good source of vital nutrients like EE and calcium [6] resulting in better performance. [16] conducted an experiment by using MOLF and found that increased body weight in treatment groups. Similarly [4] studied effect of sun dried neem leaf meal in Ross birds and found increased body weight in treatment groups.

Results showed that the treatment effect on live weight, dressed weight and dressing percent were significant (p<0.05) increasing as increase in GNH percent in feed. All organs showed significant (p<0.05) difference in their weights among treatment groups, but except heart, no one showed a definite pattern.

### Carcass quality

Statistical analysis of the value indicates significant (p<0.05) difference among different treatment groups in respect to live weight and dressed weight of birds. T4 group had the highest value and was significantly (p<0.05) higher than T2 group and T1 group while T3 and T2 group were significantly higher than T1 group. This was due to higher average body weight of treatment groups than the control group at the end of the experimental period. The results of statistical analysis revealed almost similar significant effect of GNH on dressing percentage as live weight. There was significant (p<0.05) difference between T4 group and T1 treatment group. While dressing percent of T2, T3 and T4 group was not significantly differ. This indicates that higher body weight gain of treatment groups resulting in higher dressing percent. Similar findings were reported by [4,17].

While statistical analysis indicates non-significant (p>0.05) difference among different treatment groups in respect to weights of different organs like gizzard, liver, intestine, shank except heart. Weight of heart among different treatment groups indicates that GNH have a positive effect on heart weight. This may be due higher body weight of birds in treatment groups resulting in more efficient work of the heart. Similar significant differences in heart weight were reported [4,17] by investigating the effects of MOLF and cassava leaf meal, respectively.

## Conclusion

At the end, on the basis of the performance of experimental birds in respect to feed intake, body weight, body weight gain, and FCR it seems to appear that incorporation of GNH at 6% level in the broilers ration, can successfully replace costly ingredients like maize and soybean meal in the diets of broiler birds without any harmful effects on feed intake, growth and FCR.

## Authors’ Contribution

HHS conceptualized the aim of this study, designed and supervised the experiment. NKR executed the experiments and conducted statistical analysis. SSP, DDG and MRG has drafted, corrected and revised the manuscript. APG and VKK have carried out laboratory analysis of feed samples. All authors read and approved the final manuscript.
